# Ghat: an R package for identifying adaptive polygenic traits

**DOI:** 10.1093/g3journal/jkac319

**Published:** 2022-12-01

**Authors:** Medhat Mahmoud, Mila Tost, Ngoc-Thuy Ha, Henner Simianer, Timothy Beissinger

**Affiliations:** Department of Crop Science, University of Goettingen, Goettingen 37075, Germany; Center for Integrated Breeding Research, University of Goettingen, Goettingen 37075, Germany; Department of Crop Science, University of Goettingen, Goettingen 37075, Germany; Center for Integrated Breeding Research, University of Goettingen, Goettingen 37075, Germany; Department of Animal Sciences, University of Goettingen, Goettingen 37075, Germany; Center for Integrated Breeding Research, University of Goettingen, Goettingen 37075, Germany; Department of Animal Sciences, University of Goettingen, Goettingen 37075, Germany; Department of Crop Science, University of Goettingen, Goettingen 37075, Germany; Center for Integrated Breeding Research, University of Goettingen, Goettingen 37075, Germany

**Keywords:** polygenic selection, quantitative genetics, wheat, evolution, R package, adaptation, polygenic adaptation

## Abstract

Identifying selection on polygenic complex traits in crops and livestock is important for understanding evolution and helps prioritize important characteristics for breeding. Quantitative trait loci (QTL) that contribute to polygenic trait variation often exhibit small or infinitesimal effects. This hinders the ability to detect QTL-controlling polygenic traits because enormously high statistical power is needed for their detection. Recently, we circumvented this challenge by introducing a method to identify selection on complex traits by evaluating the relationship between genome-wide changes in allele frequency and estimates of effect size. The approach involves calculating a composite statistic across all markers that capture this relationship, followed by implementing a linkage disequilibrium-aware permutation test to evaluate if the observed pattern differs from that expected due to drift during evolution and population stratification. In this manuscript, we describe “Ghat,” an R package developed to implement this method to test for selection on polygenic traits. We demonstrate the package by applying it to test for polygenic selection on 15 published European wheat traits including yield, biomass, quality, morphological characteristics, and disease resistance traits. Moreover, we applied Ghat to different simulated populations with different breeding histories and genetic architectures. The results highlight the power of Ghat to identify selection on complex traits. The Ghat package is accessible on CRAN, the Comprehensive R Archival Network, and on GitHub.

## Introduction

Many important traits in plants, animals, and humans are polygenic and depend on the cumulative effects of many loci, each contributing a small proportion of the total genetic variation (Fisher 1919). Studying the evolution and selection history of such traits can be challenging due to the small effects of the quantitative trait loci (QTL) that contribute to genetic variation. However, regardless of effect size, the theory of natural selection tells us that alleles with positive effects on fitness will tend to increase in frequency over successive generations (Wright 1931). Several statistical methods have been developed to identify loci carrying such beneficial alleles. These methods were reviewed by Ma *et al.* (2015) and include approaches relying on polymorphism variability, haplotype frequency, linkage disequilibrium (LD), allele frequency change over time, and techniques that combine some or all of these signals. However, in the case of highly polygenic traits, it is difficult or impossible to identify regions carrying individually selected alleles/haplotypes due to their small effects, regardless of which sophisticated methodologies are leveraged. For instance, it has been shown that selected loci are difficult to identify when per-locus selection intensity is low (Ma *et al.* 2015), as is the case with low heritability traits and those controlled by many alleles of small effect (Beissinger *et al.* 2018). This imposes serious limitations to the previous methods when identifying selection on polygenic traits (e.g. Lorenz *et al.* 2015).

Recently, large sample sizes, especially as utilized in human genetic studies, have allowed a more powerful understanding of the genetic architecture of polygenic traits using genome-wide association studies (GWAS) (Wood *et al.* 2014; Visscher *et al.* 2014, 2017). After conceptual work solidified the importance of polygenic adaptation (Pritchard *et al.* 2010; Pritchard and Di Rienzo 2010), researchers began placing more of an emphasis on identifying this phenomenon using an infinitesimal approach (Hancock *et al.* 2010; Berg and Coop 2014; Field *et al.* 2016; Csilléry *et al.* 2018). When polygenic adaptation is studied with an infinitesimal approach, the estimates of individual allelic effects on a phenotype are often calculated in a GWAS (Berg and Coop 2014; Berg *et al.* 2019; Sohail *et al.* 2019). The “GWAS hits” are then used to test for selection (Berg and Coop 2014; Berg *et al.* 2019; Sohail *et al.* 2019). Studies in human genetics usually use summary statistics from meta-analysis GWAS like the GIANT dataset, where many individual GWAS are combined (Turchin *et al.* 2012; Berg and Coop 2014; Racimo *et al.* 2018; Berg *et al.* 2019; Lloyd-Jones *et al.* 2019; Sohail *et al.* 2019). These studies observed strong signals for polygenic selection (Turchin *et al.* 2012; Berg and Coop 2014; Racimo *et al.* 2018; Berg *et al.* 2019; Lloyd-Jones *et al.* 2019; Sohail *et al.* 2019). The observed signals did not replicate when the estimates of individual allelic effects were calculated based on GWAS conducted in the unstructured UK Biobank dataset, especially when the dataset was filtered for individuals with a common ancestry (Berg *et al.* 2019; Sohail *et al.* 2019). Apparently estimates of allelic effects derived from GWAS are affected by population confounding effects, and when they are combined in polygenic scores, they are prone to bias due to misestimation of effects (Racimo *et al.* 2018; Berg *et al.* 2019; Sohail *et al.* 2019). Furthermore, Berg *et al*. (2019) observed that methods like principal component analysis (PCA) do not properly accounting for population confounding effects.

Zeng *et al*. (2018) developed a Bayesian mixed linear model (BayesS) based on the relationship between SNP effect sizes and minor allele frequency. This approach uses genomic data and fits all SNP effects together as random effects (Zeng *et al.* 2018). The identified signals of polygenic selection from Zeng *et al*. (2018) conflict with the results from Berg *et al*. (2019), even though both studies worked with the unstructured UK Biobank dataset. Berg *et al*. (2019) filtered the samples based on British ancestry, whereas Zeng *et al*. (2018) included samples of all European ancestry, which might suggest that residual European population structure continues to be confound, even when advanced methods for corrections for population structure were applied (Berg *et al.* 2019). On the other hand, BayesS used by Zeng *et al*. (2018) is based on the relationship between SNP effect sizes and minor allele frequency, whereas minor allele frequency and SNP effects might correlate due to estimation error during genomic prediction (Beissinger *et al.* 2018).

The previously mentioned approaches were developed for human genetics, and most of these approaches are based on summary statistics from massive GWAS which are not always feasible for other species, e.g. due to budgetary limitations. Josephs *et al*. (2019) developed a statistic, referred as conditional *Q*_PC_, which detects adaptive divergence while controlling for population substructure. Conditional *Q*_PC_ detects adaptive divergence based on the excess of variation observed among populations versus variation observed within populations (Josephs *et al.* 2019). The underlying assumption of this test is that adaptive divergence among populations increases the trait variance explained by the first principal components (PCs) relative to the later PCs (Josephs *et al.* 2019). The PCs are computed based on the relatedness matrix, therefore the conditional *Q*_PC_ detects substructures that are associated with trait divergence, and based on these observations, conclusions about polygenic adaptation regarding the trait are drawn (Josephs *et al.* 2019).

Another test, denoted $\hat{G}$ (or Ghat), has been developed to identify selection on polygenic traits using whole-genome data (Beissinger *et al.* 2018). Ghat combines information from all loci simultaneously to evaluate whether there is a trend for loci contributing to a phenotype to collectively show evidence of selection (Beissinger *et al.* 2018). Thereby, signals that are individually insignificant for selection (at the locus level) may become highly significant when evaluated at the genome-wide scale (at the level of finding selection on a trait). Furthermore, because Ghat uses pre- and post-selection data, it can identify polygenic selection within shorter subperiods of time and compare these to each other (Beissinger *et al.* 2018). Another advantage of Ghat is that it is based on the relationship between SNP effects and allele frequency change over time at every locus, which should not correlate unless there has been on-going selection in comparison to minor allele frequency and SNP effects (Beissinger *et al.* 2018; Zeng *et al.* 2018).

We have developed and released an R (“R: The R Project for Statistical Computing”) package called “Ghat” to implement the $\hat{G}$ test. The package allows users to test for selection and evolution in quantitative traits, and as shown by Beissinger *et al*. (2018), it is particularly powerful when testing for selection on highly polygenic traits. To evaluate and demonstrate the package in this manuscript, we applied the method and corresponding Ghat package to 15 different traits measured in a winter wheat collection of 191 cultivars, registered mainly in Western Europe between 1966 and 2013, which were published by Voss-Fels *et al*. (2019). We then applied Ghat package on a simulated cattle population to validate the package and explore different situations where it is most and least powerful.

## Implementation of the Ghat package

### Overview

Ghat identifies selection by leveraging all genotyped loci simultaneously (Beissinger *et al.* 2018). This makes it possible to identify selection in complex traits that are controlled by many genes with small effects (Beissinger *et al.* 2018). First, all allelic effects are estimated using a genomic prediction approach. Next, allele frequencies are estimated in 2 or more different generations (e.g. generations 0 and 10) (Beissinger *et al.* 2018). Third, the rate of LD decay is estimated to approximate the number of independent genome segments in the study population (Beissinger *et al.* 2018). Finally, an LD-aware permutation test is implemented to evaluate if there is evidence of significant selection between generations. The direction of selection is calculated based on the relationship between the change in allele frequency between the generations and the estimated additive effect (Beissinger *et al.* 2018). Complete theoretical and methodological details are provided by Beissinger *et al.* (2018).

### Installation

**Fig. 1.**
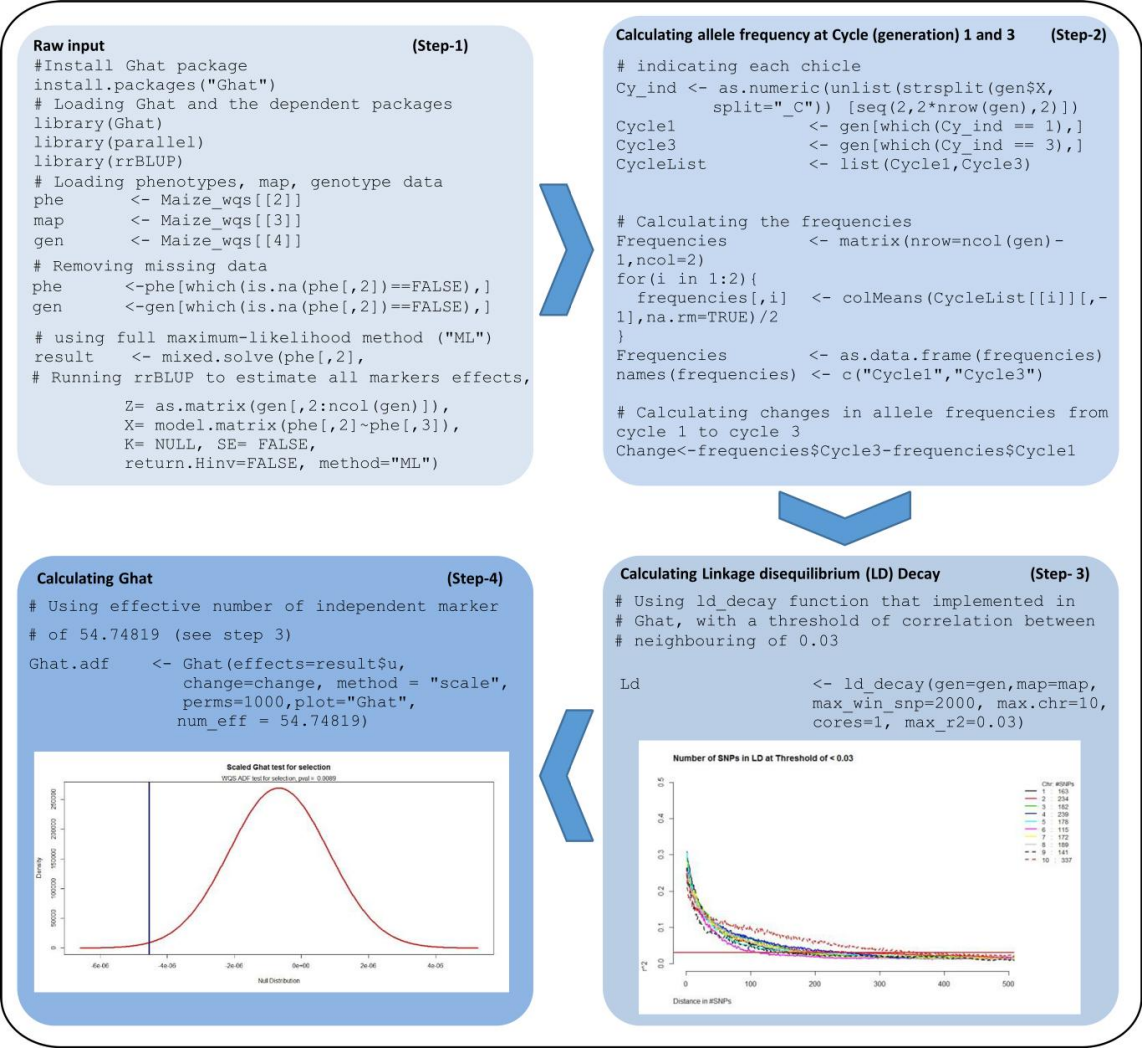
Four steps to go from raw individual marker data to results from the Ghat test for selection. An illustrated example using the “Maize_wqs” data available in the Ghat package. Step-1: install the package, load the dependencies and “Maize_wqs” data, and estimate allele substitution effects (e.g. by using rrBLUP; Endelman 2011). Step-2: calculate the differences in allele frequency between 2 different generations (cycle 1 and cycle 3). Step-3: estimate the decay of LD to calculate the effective number of independent markers. Step-4: calculate the Ghat value and *P*-value from the permutation test.

The Ghat package is available on the Comprehensive R Archival Network (CRAN) (Mahmoud *et al.* 2019; “R: The R Project for Statistical Computing”) and can be installed within the R terminal (Fig. 1, step-1). The package requests the following optional R-package dependencies:

- Parallel-Package {parallel}: Support for parallel computation, to speed the computation time in large data analysis (Ooi *et al.* 2019).
- rrBLUP-Package {rrBLUP}: Ridge regression for estimating marker effects (RR-BLUP) (Endelman 2011).

These may be installed and loaded before running Ghat by typing: >install.packages(c(“parallel”, “rrBLUP”)) and then typing > library(parallel) and > library(rrBLUP) (Fig. 1, step-1).

### Input data format

For its simplest implementation, Ghat requires only 2 vectors of input. The first of these is a vector of allele substitution effects, and the second is a vector containing the allele frequency change between the 2 generations (Supplementary Table 1) (Beissinger *et al.* 2018). Required parameters are, “method”: to specify how the Ghat permutation test should account for LD between markers (“scale” is usually the most appropriate method to implement because it accounts for LD between markers, as described below), “perms”: for the number of permutations to perform, “plot”: to specify whether or not simple Ghat plots should be output, “blocksize”: for setting the size of blocks that should be used for LD-trimming (only required when method = “trim”), and “num_eff”: for setting the effective number of independent segments of the genome (only required when method = “scale”). All parameter details are described in Supplementary Table 1.

### Establishing the number of effective markers

Treating each marker as an independent observation can lead to overestimated test statistics. This is because allele frequencies and effect estimates from markers that are in LD are not independent (Beissinger *et al.* 2018). Any level of LD between markers leads to fewer independent observations than the total number of markers (Beissinger *et al.* 2018). To avoid such inflated signal of polygenic adaptation, we include an option in Ghat package called “scale,” which scales the variance of the permutation test statistic according to the realized extent of LD (Beissinger *et al.* 2018). More details on the statistical background of the scaling method were published by Beissinger *et al*. (2018). In the Ghat package, we provide a function called “ld_decay” that can estimate the LD decay across the entire genome and uses this information to approximate the total number of independent genome segments, or “genotype blocks” (Beissinger *et al.* 2018). The following command runs the ld_decay function >Ld < - ld_decay(gen, map, max_win_snp, max.chr, cores = 1, max_r2) (Fig. 1, step-3) (Beissinger *et al.* 2018). The output information is used by Ghat to scale the variance of the permutation test statistic according to the actual number of independent markers (Fig. 1, step-4) (Beissinger *et al.* 2018).

### Running the test

To run Ghat, estimates of allele substitution effects and allele frequency changes between 2 generations (or populations) are required (Beissinger *et al.* 2018). Next, to perform the Ghat test with this information, only 1 line of code is required: >Ghat_res < - Ghat(effects, change, method, perms, plot, num_eff) (Fig. 1, step-4) (Beissinger *et al.* 2018). All of the functions, options, and datasets available in Ghat are provided in Supplementary Table 1.

## Part I: testing for selection during 50 years of wheat breeding

To demonstrate the power and flexibility of the Ghat R package, we used it to test for selection during the past 50 years of wheat breeding in Western Europe. To achieve this, we leveraged a dataset representing 50 years of commercial winter wheat varieties that were previously published by Voss-Fels and colleagues (Voss-Fels *et al.* 2019). Our analysis methods and results are described below.

### Methods

#### Genotypes and phenotypes

A dataset of 191 cultivars, registered mainly in Western Europe between 1966 and 2013, was previously published by Voss-Fels and his group (Voss-Fels *et al.* 2019). In total, 19 traits were considered and used to evaluate and quantify the phenotypic progress of wheat breeding during the last 5 decades. The cultivars in the dataset are mainly registered and bred for the German market (Voss-Fels *et al.* 2019).

After filtering for data quality, 15 traits remained in the dataset. Spikes per sqm, heading, sedimentation, falling number, and green canopy duration were removed from the analysis because of missing phenotypic information (>70% of total phenotypes were missing). All traits were tested in 3 different environments by Voss-Fels *et al*. (2019). The environments were: (1) a high-intensity nitrogen supply along with best-practices fungicide, insecticide, and growth regulator applications (HiN/HiF treatment), (2) a high level of nitrogen fertilization with a fungicide-free treatment (HiN/NoF), and (3) a low level of nitrogen fertilization with a fungicide-free treatment (LoN/NoF). The evaluation was based on 5 main groups of traits: (1) *yield parameter traits*, including grain yield, harvest index, radiation use efficiency, and radiation interception efficiency; (2) *biomass parameter traits*, including above-ground biomass, kernels per sqm, and thousand-kernel weight; (3) *quality parameter traits*, including crude protein, protein yield, and nitrogen use efficiency; (4) *morphological traits*, including plant height, green canopy duration, and kernel per spike; and (5) *disease resistance traits*, including resistance to powdery mildew and resistance to stripe rust. All cultivars were genotyped with a 15K SNP Illumina Infinium iSelect genotyping array (Muqaddasi *et al.* 2017) by Voss-Fels *et al*. (2019). After filtering, 8,710 SNPs passed the genotype quality control criteria (Voss-Fels *et al.* 2019) and were included in their subsequent analyses. In our analysis, we re-analyzed the exact set of SNPs that were published by Voss-Fels *et al*. (2019), as described above. All heritability estimates and genetic correlations between the same traits measured in different environments were taken from the aforementioned publication (Voss-Fels *et al.* 2019).

#### Phenotypic progress

To create a baseline for progress that we could use to compare against Ghat results, we re-estimated the phenotypic progress of the 15 wheat traits over 50 years of selection and adaptation using simple *Pearson* correlations between phenotype and the year of release (Benesty *et al.* 2009).

#### Ghat

Allelic effects at every marker locus for the 15 traits were estimated using rrBLUP (Endelman 2011), and changes in allele frequencies were calculated with R (“R: The R Project for Statistical Computing”). Ghat is calculated as the summation of the estimated allele frequency change of every SNP multiplied by its effect size, according to

(1) $$\hat{G}=\overset{m}{\underset{\;j=1}{\sum }}\;\Delta_{j}\alpha_{j}$$

where Δ_*j*_ is the frequency change in locus *j* between 2 time points and *α*_*j*_ is the additive effect of locus *j.* We implemented 2 different analyses with Ghat to test for selection, which enabled us to measure the efficiency of the test in different situations. In the first analysis, which we call “All Phenotypes,” allelic effects were estimated using phenotypic information from all available cultivars in the Voss-Fels *et al*. (2019) study, which represent the last 50 years of wheat breeding. This represents the best-case Ghat analysis, in which all phenotypic information is available. In the second analysis, which we call “Modern Phenotypes,” allelic effects were estimated using only modern phenotypic information (from 2010 to 2013), while changes in allele frequency were still calculated based on genotypes from opposite ends of the 50-year dataset. This represents a realistic use-case for Ghat, when all genotypic information is available, but phenotypic information is only available for a subset of the individuals (e.g. from modern germplasm). In other words, this mimics the situation where reliably measured historical phenotypes do not exist, but historical tissues or seeds are available and can be used for DNA collection.

#### Principal component analysis

PCA was performed with an R (Jombart 2008; “R: The R Project for Statistical Computing”) package called “adegenet,” which was developed for multivariate analysis of genetic markers (Jombart 2008). The PCA was conducted with all 191 cultivars with 8,710 markers to detect the presence of population structure. For this analysis, missing data were imputed according to the mean allele frequency (Jombart *et al.* 2010).

### Results

#### Testing for selection during 50 years of wheat breeding

The main theory behind this test for polygenic selection is that alleles at every locus, distributed across the entire genome, may contribute a small proportion of the total variance for each trait. Therefore, previous studies either required large sample sizes (Lorenz *et al.* 2015) or were unsuccessful in identifying the signals of selection on a genetic level (Lorenz *et al.* 2015). To demonstrate the Ghat package, we used it to test for selection on the 15 wheat traits, enabling us to establish how much power is lost when phenotypic data are only available from modern lines.

#### Phenotypic progress

**Fig. 2.**
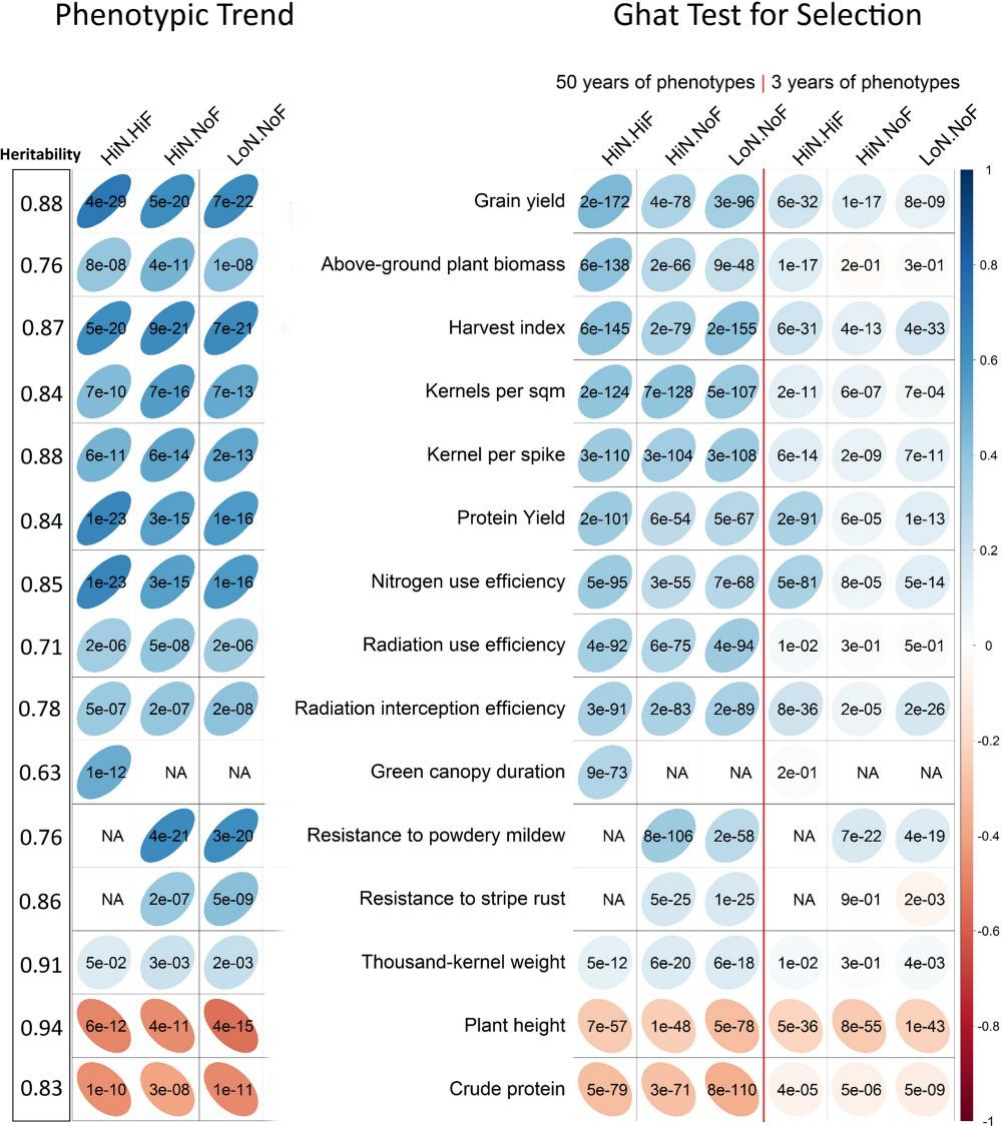
Assessment of **G**hat when applied to European winter wheat under 3 agrochemical treatments: (1) a high-intensity nitrogen supply along with best-practices fungicide, insecticide, and growth regulator applications (HiN/HiF), (2) a high level of nitrogen fertilization with a fungicide-free treatment (HiN/NoF), and (3) a low level of nitrogen fertilization with a fungicide-free treatment (LoN/NoF). Left: the phenotypic trend over the last 50 years. Right: The Ghat test of selection based on 2 analyses. “All Phenotypes” refers to estimating allelic effects using all available phenotypic information over the 50 years of wheat breeding; “Modern Phenotypes” refers to estimating allelic effects using modern phenotypic information only (2010–2013). Large light ellipses correspond to weaker selection intensities; thin dark ellipse correspond to more selection intensity. *P*-values printed on each cell on the left side of the figure (phenotypic trend section) correspond to the significance of the phenotypic trend. *P*-values printed on each cell on the right side of the figure (Ghat test section) correspond to implementing the Ghat permutation test.

After estimating the phenotypic trend (correlation between phenotypic value and the registration year) (Fig. 2), we can classify the 15 traits into 3 groups. The first group is showing comprised traits that increased in value during breeding. These were above-ground plant biomass, grain yield, green canopy duration, harvest index, kernels per spike, kernels per square meter, nitrogen use efficiency, protein yield, radiation interception efficiency, radiation use efficiency, resistance to powdery mildew, and resistance to stripe rust. Next, we identified a group of traits with a decreasing phenotypic trend, which was composed of plant height and crude protein. The last group contained traits without a strong overarching phenotypic trend. Only thousand-kernel weight was in this group. The correlation for thousand-kernel weight was positive but relatively weak in comparison correlations observed in the first group (Fig. 2).

#### Ghat test for selection

Figure 2 shows the results of the Ghat test when testing for selection between 1966 and 2013 in winter wheat breeding programs from mainly Western Europe. First, we investigated the precision and accuracy of the Ghat test by estimating the significance, and direction of selection and comparing the results to phenotypic progress over a time frame of 50 years. Overall, the selection size and direction on the 15 traits using all phenotypes were in the strong agreement with the phenotypic trend. Next, we evaluated the results from Ghat using phenotypic information from only the most recent 3 years of the study (but all genotypic data were still included). Notably, this was also in a strong agreement with the phenotypic trend. Ghat successfully detected the signals of selection from the past in each trait, even when only modern phenotypes were utilized. For example, the results from the Ghat test indicated strong, directional selection for plant height and crude protein traits, which agrees with their phenotypic trends during the last 50 years (Voss-Fels *et al.* 2019). Ghat showed very weak selection for thousand-kernel weight, which again aligned with this trait's observed phenotypic trend (Voss-Fels *et al.* 2019). The rest of the traits were under high to moderate directional selection. To validate the results of Ghat on wheat data, *spearman* correlations between the Ghat results from 3 and 50 years of phenotypes were estimated. A significant (*r* = 0.57; *P* < 0.001) correlation was found between Ghat values from 3 and 50 years of phenotypes, indicating that even in the absence of historical phenotypic information, Ghat can be used to understand historical patterns of selection.

#### Assessing population stratification

**Fig. 3.**
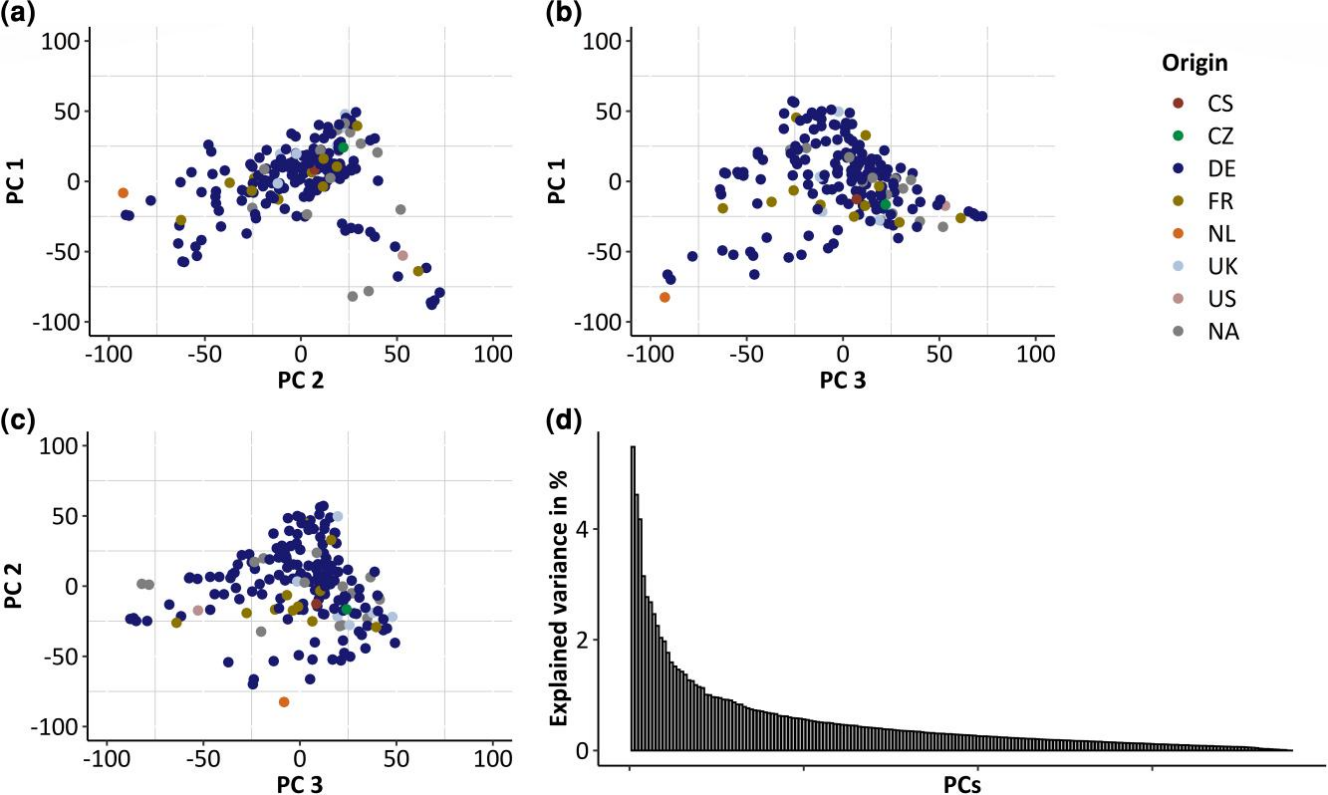
PCA: a) PC1 plotted against PC2; b) PC1 plotted against PC3; c) PC2 plotted against PC3; and d) the eigenvalues of all the detected PCs.

Voss-Fels *et al*. (2019) observed weak or no population stratification, and explain that this observation may be caused by a large germplasm exchange between breeding companies. We re-analyzed the data using PCA and could identify no major groupings (Fig. 3). In the PCA, the first 3 PCs explain together 16.05% of the variance, which do not correspond to their geographic location (Fig. 3). However, no analysis can disprove that cryptic population stratification may be present in a dataset. This analysis was performed because excessive population stratification can cause erroneous signals of selection to be identified (Berg *et al.* 2019).

#### Adaptation history of Western European winter wheat

**Fig. 4.**
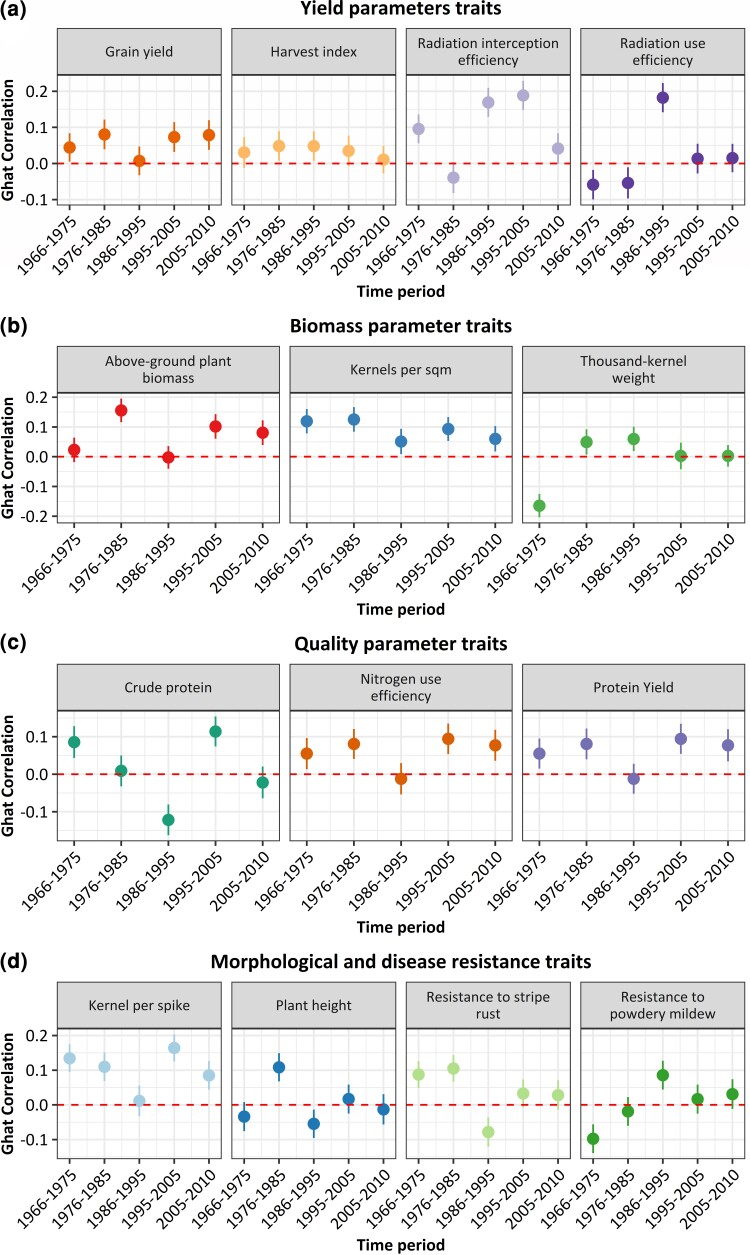
Ghat correlation estimates of the selection size and direction for 50 years of wheat breeding. Results are for 14 productivity traits in winter wheat from Western Europe measured during the past 50 years. Points above zero level (dashed lines) indicate positive selection, while points under zero indicate negative selection; bold lines represent 99% confidence interval. a) Adaptation history for the yield parameter traits. b) Adaptation history for the biomass parameter traits. c) Adaptation history for quality parameter traits. d) Adaptation history for morphological and disease resistance traits.

To dissect the history of selection in wheat more precisely, we divided the 50 years of breeding into 10 5-year time periods and applied the Ghat test successively for each period. We excluded the green canopy duration trait from this analysis since it was only measured under 1 environmental condition. For the remaining 14 traits, we calculated the average phenotypic value across the 3 different environmental conditions. For example, during the period from 1966 to 1975, significantly selected traits according to the Ghat test included grain yield, harvest index (Fig. 4a), above-ground plant biomass, thousand-kernel weight (Fig. 4b), crude protein, nitrogen use efficiency (Fig. 4c), kernel per spike and resistance to stripe rust (Fig. 4d). However, during the same period, selection operated significantly in the negative direction against radiation use efficiency (Fig. 4a), thousand-kernel weight (Fig. 4b), plant height and resistance to powdery mildew (Fig. 4d). Therefore, by considering the single time periods of selection within the past 50 years (Fig. 4), and regarding the traits which were selected within the different time periods, we can deduce information about the focus of the breeders during that time period or the environmental conditions during the time period. The number of cultivars available for the 10 adaptation periods is as follows: 18 cultivars from 1966 to 1975; 19 cultivars from 1976 to 1985; 25 cultivars from 1986 to 1995; 48 cultivars from 1996 to 2005; 81 cultivars from 2006 to 2015.

## Part II: assessment of Ghat to identify selection in a simulated divergently selected cattle population

To complement the above wheat analysis, we additionally generated a simulated cattle dataset with divergent selection to further test and demonstrate the efficacy of Ghat. This allowed us to evaluate how the Ghat package performs in terms of different genetic architectures and experimental parameters, which are never perfectly known using real data. In this analysis, we assessed the impact of trait heritability (*h^2^*), sample size (*n*), trait polygenicity [number of QTL (*n*QTL)], and marker density (MD) on the performance of Ghat.

### Methods

#### Data simulation

We simulated 30 bovine chromosomes of a length of 100 cM using QMsim (Sargolzaei and Schenkel 2009). Our simulations started with 100 historical generations of drift-only, and then we sampled 2 distinct populations to undergo selection (2 isolated populations). Population-A (unselected population) is a population that underwent complete random mating (i.e. no selection) for 20 generations; Population-B (selected population) is a population that underwent strong phenotypic selection for 20 generations (Supplementary Fig. 1). Aside from selection, Population-A and Population-B were identical and were treated identically during the simulated experiment. After simulating the populations, we applied Ghat to test for selection during the most recent 5 generations (generation #20–generation #15) in both populations, creating a realistic situation in dairy cattle breeding. We estimated the allele effects using the genotypes of all 20 generations for more accurate estimation. Supplementary Fig. 1 depicts this simulation scheme.

Four groups of datasets were simulated for studying the performance of Ghat (Table 1). Each group involved the creation of different datasets simulated with 3 constants parameters and 1 varying parameter (i.e. the parameter under investigation). Group 1 was simulated to investigate the effects of *n* in the Ghat test; we simulated 10 datasets with a constant *h*^2^, nQTL, and MD, but with a different level of *n* (from 50 to 25,600 animals) in each dataset. Then we measured the *P* values coming from using these different datasets with different *n*. Group 2 was simulated to investigate the effect of *h*^2^ on Ghat performance. For this, we simulated 10 datasets with a constant *n*, *n*QTL, and MD, but with a different *h*^2^ (from 0.1 to 0.95) in each dataset. Group 3 investigated the effect of *n*QTL on Ghat performance. For group 3, we simulated 10 datasets with a constant *n*, *h*^2^, and MD, but with a different level of *n*QTL (from 30 to 15,360 QTL) in each dataset. Group 4 was simulated to investigate the effect of MD on Ghat performance. In this group, we simulated 10 datasets with a constant *n*, *h*^2^, and *n*QTL but with a different level of MD (from 30 to ∼ 1 million markers) in each dataset.

**Table 1.** Presentation of all datasets used in the Ghat test.

Dataset	To investigate	Sample size	Heritability	QTL number	Marker density
Group 1	Sample size	50:25,600*^a^*	0.5	150	10,020
Group 2	Heritability	400	0.1:0.95*^b^*	150	10,020
Group 3	QTL number	400	0.5	30:15,360*^c^*	10,020
Group 4	Marker density	400	0.5	150	30:1 million*^d^*

Sample size (*n*): 50, 100, 200, 400, 800, 1,600, 3,200, 6,400, 12,800, and 25,600 animals.

Heritability (*h*^2^): 0.1, 0.2, 0.3, 0.4, 0.5, 0.6, 0.7, 0.8, 0.9, and 0.95 of *h*^2^.

QTL number (*n*QTL): 30, 60, 120, 240, 480, 960, 1,920, 3,840, 7,680, and 15,360 QTL.

Marker density (MD): 30, 300, 3k, 30k, 60k, 120k, 240k, 480k, 960k, and ∼1 million markers.

#### Ghat implementation

Genomic prediction methods like BayesR, BayesB, and BayesC assume that the allele substitution effects follow a distribution in which many effects are zero and some have moderate effects, subsequently these methods may for some genetic architectures lead to higher accuracy than the GBLUP/rrBLUP methods (Meuwissen *et al.* 2013). Since most of the bovine traits were simulated with a large number of QTL of small effect, allele substitution effects at every marker locus were estimated using BayesC (Pérez and de los Campos 2014). The effective number of independent marker genome segments was estimated using SimpleM algorithm in R (Gao *et al.* 2008; “R: The R Project for Statistical Computing”), and changes in allele frequencies were calculated with R. All R-code is publicity available on GitHub (https://github.com/Medhat86/Selection-and-adaptation-on-simulated-bovine-genome).

Ghat is calculated as the summation of the estimated effect of every SNP scored multiplied by its effect size, according to model 1. We implemented identical analyses on selected and unselected populations across 1,000 simulation replications for 4 different tests (*n*, *h*^2^, *n*QTL, and MD) to evaluate the detection accuracy by evaluating true discovery rates and false positives. Confidence intervals were obtained from *P*-values of the same traits from the Ghat permutation test using the formula from Bland and Altman (1986).

### Results

#### The effect of sample size on Ghat

Based on 1,000 replications, Supplementary Fig. 2a shows the impact of *n* on the performance of Ghat through quantifying the *P*-values of the Ghat test for selection. When we test the unselected population with a constant *h*^2^ = 0.5, *n*QTL = 150 and 10,020 markers, 99% of replicated traits were not significantly (*P*-value < 0.01) under selection. Conversely, in the selected population 99.3% of replicated traits found highly significant under selection (*P*-value < 0.01). The *P*-value also shows a decreasing trend with increasing *n* (from 50 to 25,600 animals) in the selected population. Moreover, the confidence interval decreases with increasing *n*.

#### The effect of heritability on Ghat

Based on 1,000 replications, Supplementary Fig. 2b shows the impact of *h*^2^ on the power of Ghat. Higher *h*^2^ led to a more powerful test. When we tested the unselected population with a constant *n* = 400, *n*QTL = 150 and 10,020 markers, 100% of replicates were not significantly (*P*-value < 0.01) under selection. In the selected populations 100% of replicates were significant for selection (*P*-value < 0.01). The *P*-value shows a consistency decrease with increasing trait *h*^2^ (from 0.1 to 0.95 of *h*^2^).

#### The effect of the number of QTL controlling a trait on Ghat

Based on 1,000 replicates, Supplementary Fig. 2c shows the impact of *n*QTL on the power of Ghat. When we tested the unselected population with a constant *n* = 400, *h*^2^ = 0.5 and 10,020 markers, 99.7% of replicated traits were not significantly (*P*-value < 0.01) under selection. In the selected population, 100% of replicated traits found highly significant selection (*P*-value < 0.01). The power of the test increased with increasing the *n*QTL in the selected population. This can be restated to emphasize that, all else being equal, Ghat is more able to detect selection on traits that are more complex (i.e. controlled by a large number of QTL).

#### The effect of MD on Ghat

Based on 1,000 replications, Supplementary Fig. 2d shows the impact of MD on the performance of Ghat through quantifying the *P*-values of the Ghat test for selection. When we tested the unselected population with a constant *n* = 400, *h*^2^ = 0.5 and 150 QTL, 90% of replicated traits were not significantly (*P*-value < 0.01) under selection. While, in the selected population 100% of replicated traits were highly significant for selection (*P*-value < 0.01). The *P*-value also shows an inconsistent increase of significance along with an increasing MD in the selected population (from 30 to ∼ 1 million MD). This may be caused by multicollinearity in the data when there are a high number of markers (>120,000 MD) and relatively small n (400 animals).

#### Testing and system requirements

We have released Ghat package on CRAN (https://cran.r-project.org/package=Ghat) for the 3 major operating systems: MS Windows, Linux/Unix, and Mac OS. We have also released the source code on GitHub (https://github.com/Medhat86/Ghat) so that users can compile for specific platforms or modify the code as needed.

For users who have already computed allele effect and allele frequencies changes and are applying the Ghat test to these data, the computational cost of Ghat is unnoticeable. Practically, estimating Ghat for maize data [5] with 1,000 permutations takes 1 s per 1 phenotype on a 2019 Dell XPS 23 with 2.4 Intel Core i7 processor.

## Discussion and future directions

As shown herein, the Ghat package can offer high power to detect selection on polygenic traits. Ghat identified polygenic selection on several candidate traits which were actually selected in winter wheat breeding programs. However, the wheat dataset included a relatively small number of markers (8,710) relative to current high-density marker panels and/or whole-genome sequencing approaches, so this analysis did not represent all the challenges associated with implementing Ghat in these settings. In particular, high-density panels include markers that are in strong LD, which must be properly accounted for in any Ghat implementation.

To address this, we used a simulated SNP panel to further explore the utility of Ghat when high LD between markers is present. We found that Bayesian methods, such as BayesC (Habier *et al.* 2011; Meuwissen *et al.* 2013), as implemented here, can be useful because of their ability to employ feature selection for marker number reduction (Habier *et al.* 2007, 2010, 2011). BayesC does this by assuming that some fractions of the markers have zero effect on the trait of interest and forcing all other effects to equal zero. While Ghat proved to be powerful when paired with BayesC, Ghat is flexible and able to use marker effect estimates extracted from any prediction method. Further research should be conducted to determine the ideal feature-selection approaches to pair with Ghat.

Unlike methods for detecting selected loci, Ghat can quantify the direction of selection even in the absence of phenotypes from a preselection population. For breeders and breeding organizations, this represents a practical solution for tracing selection and adaptation history based on phenotypes measured from modern germplasm coupled with DNA samples saved from the past. From an economical perspective, this may allow a retrospective evaluation of traits that were inadvertently selected in the past, and therefore may productively serve as the targets of intentional selection in the future. Advances in phenotyping technologies make measuring novel traits increasingly possible (Cobb *et al.* 2013; Mahmoud *et al.* 2017), and Ghat allows researchers to understand selection on traits that could not be measured previously for practical or technological reasons.

The Ghat test and implementation via the package described herein does not require immense sample size, individual genotypes, and/or genome-wide significant SNPs, as demonstrated by our implementation with a relatively modest sample size of 191 wheat individuals genotyped for 8,710 SNPs. Notably, this approach is more powerful when traits are controlled by a large number of QTLs (Beissinger *et al.* 2018). These advantages allow the Ghat package to be used for identifying selection for various complex traits in other species of plants, animals, and humans.

## Supplementary Material

jkac319_Supplementary_Data

## Data Availability

The Ghat R package described in this manuscript is open source and available on CRAN (https://cran.r-project.org/package=Ghat). All analysis and simulation scripts used in this manuscript are available on GitHub (https://github.com/Medhat86/Ghat & https://github.com/Medhat86/Selection-and-adaptation-on-simulated-bovine-genome). The wheat dataset we utilized was previously published and released by Voss-Fels *et al.* (2019). Supplemental material available at G3 online.

## Notes

*Communicating editor: G. de los Campos*

## References

[jkac319-B1] Beissinger T , Kruppa J, Cavero D, Ha N-T, Erbe M, Simianer H. A simple test identifies selection on complex traits. Genetics. 2018;209(1):321–333. doi:10.1534/genetics.118.300857. 29545467 PMC5937188

[jkac319-B2] Benesty J , Chen J, Huang Y, Cohen I. Pearson correlation coefficient. Noise reduction in speech processing, Springer topics in signal processing. Berlin (HDB): Springer Berlin Heidelberg; 2009. p. 1–4.

[jkac319-B3] Berg JJ , Coop G. A population genetic signal of polygenic adaptation. PLoS Genet. 2014;10(8):e1004412. doi:10.1371/journal.pgen.1004412. PMC4125079 25102153

[jkac319-B4] Berg JJ , Harpak A, Sinnott-Armstrong N, Joergensen AM, Mostafavi H, Field Y, Boyle EA, Zhang X, Racimo F, Pritchard J, Coop G. Reduced signal for polygenic adaptation of height in UK Biobank. eLife. 2019;8:e39725. doi:10.7554/eLife.39725. 30895923 PMC6428572

[jkac319-B5] Bland MJ , Altman DG. Statistical methods for assessing agreement between two methods of clinical measurement. Lancet. 1986;327(8476):307–310. doi:10.1016/S0140-6736(86)90837-8. 2868172

[jkac319-B6] Cobb JN , DeClerck G, Greenberg A, Clark R, McCouch S. Next-generation phenotyping: requirements and strategies for enhancing our understanding of genotype–phenotype relationships and its relevance to crop improvement. Theor Appl Genet. 2013;126(4):867–887. doi:10.1007/s00122-013-2066-0. 23471459 PMC3607725

[jkac319-B7] Csilléry K , Rodríguez-Verdugo A, Rellstab C, Guillaume F. Detecting the genomic signal of polygenic adaptation and the role of epistasis in evolution. Mol Ecol. 2018;27(3):606–612. doi:10.1111/mec.14499. 29385652

[jkac319-B8] Endelman JB . Ridge regression and other kernels for genomic selection with R package rrBLUP. Plant Genome. 2011;4(3):250–255. doi:10.3835/plantgenome2011.08.0024.

[jkac319-B9] Field Y , Boyle EA, Telis N, Gao Z, Gaulton KJ, Golan D, Yengo L, Rocheleau G, Froguel P, McCarthy MI, Prtichard J. Detection of human adaptation during the past 2000 years. Science. 2016;354(6313):760–764. doi:10.1126/science.aag0776. 27738015 PMC5182071

[jkac319-B10] Fisher RA . XV.—the correlation between relatives on the supposition of Mendelian inheritance. Earth Environ Sci Trans R Soc Edinb. 1919;52(2):399–433. doi:10.1017/S0080456800012163.

[jkac319-B11] Gao X , Starmer J, Martin ER. A multiple testing correction method for genetic association studies using correlated single nucleotide polymorphisms. Genet Epidemiol. 2008;32(4):361–369. doi:10.1002/gepi.20310. 18271029

[jkac319-B12] Habier D , Fernando RL, Dekkers JCM. The impact of genetic relationship information on genome-assisted breeding values. Genetics. 2007;177(4):2389–2397. doi:10.1534/genetics.107.081190. 18073436 PMC2219482

[jkac319-B13] Habier D , Fernando RL, Kizilkaya K, Garrick DJ. Extension of the Bayesian alphabet for genomic selection. BMC Bioinformatics. 2011;12(1):186. doi:10.1186/1471-2105-12-186. 21605355 PMC3144464

[jkac319-B14] Habier D , Tetens J, Seefried F-R, Lichtner P, Thaller G. The impact of genetic relationship information on genomic breeding values in German Holstein cattle. Genet Sel Evol. 2010;42(1):5. doi:10.1186/1297-9686-42-5. 20170500 PMC2838754

[jkac319-B15] Hancock AM , Alkorta-Aranburu G, Witonsky DB, Di Rienzo A. Adaptations to new environments in humans: the role of subtle allele frequency shifts. Philos Trans R Soc B Biol Sci. 2010;365(1552):2459–2468. doi:10.1098/rstb.2010.0032. PMC2935101 20643735

[jkac319-B16] Jombart T . Adegenet: a R package for the multivariate analysis of genetic markers. Bioinformatics. 2008;24(11):1403–1405. doi:10.1093/bioinformatics/btn129. 18397895

[jkac319-B17] Jombart T , Devillard S, Balloux F. Discriminant analysis of principal components: a new method for the analysis of genetically structured populations. BMC Genet. 2010;11(1):94. doi:10.1186/1471-2156-11-94. 20950446 PMC2973851

[jkac319-B18] Josephs EB , Berg JJ, Ross-Ibarra J, Coop G. Detecting adaptive differentiation in structured populations with genomic data and common gardens. Genetics. 2019;211(3):989–1004. doi:10.1534/genetics.118.301786. 30679259 PMC6404252

[jkac319-B19] Lloyd-Jones LR , Zeng J, Sidorenko J, Yengo L, Moser G, Kemper K, Wang H, Zheng Z, Magi R, Esko T, Metspalu A, Wray N, Goddard ME, Yang J, Visscher P. Improved polygenic prediction by Bayesian multiple regression on summary statistics. Nat Commun. 2019;10(1):5086. doi:10.1038/s41467-019-12653-0. 31704910 PMC6841727

[jkac319-B20] Lorenz AJ , Beissinger TM, Silva RR, de Leon N. Selection for silage yield and composition did not affect genomic diversity within the Wisconsin quality synthetic maize population. G3 (Bethesda). 2015;5(4):541–549. doi:10.1534/g3.114.015263. 25645532 PMC4390570

[jkac319-B21] Ma Y , Ding X, Qanbari S, Weigend S, Zhang Q, Simianer H. Properties of different selection signature statistics and a new strategy for combining them. Heredity (Edinb). 2015;115(5):426–436. doi:10.1038/hdy.2015.42. 25990878 PMC4611237

[jkac319-B22] Mahmoud M , Ha N-T, Beissinger T. Ghat: Quantifying Evolution and Selection on Complex Traits. 2019. https://cran.r-project.org/web/packages/Ghat/index.html.

[jkac319-B23] Mahmoud M , Yin T, Brügemann K, König S. Phenotypic, genetic, and single nucleotide polymorphism marker associations between calf diseases and subsequent performance and disease occurrences of first-lactation German Holstein cows. J Dairy Sci. 2017;100(3):2017–2031. doi:10.3168/jds.2016-11767. 28109590

[jkac319-B24] Meuwissen T , Hayes B, Goddard M. Accelerating improvement of livestock with genomic selection. Annu Rev Anim Biosci. 2013;1(1):221–237. doi:10.1146/annurev-animal-031412-103705. 25387018

[jkac319-B25] Muqaddasi QH , Brassac J, Börner A, Pillen K, Röder MS. Genetic architecture of anther extrusion in spring and winter wheat. Front Plant Sci. 2017;8:754. doi:10.3389/fpls.2017.00754. 28559904 PMC5432570

[jkac319-B26] Ooi H , Corporation M, Weston S, Tenenbaum D. *doParallel: Foreach Parallel Adaptor for the “parallel” Package*, 2019. https://cran.r-project.org/web/packages/doParallel/index.html.

[jkac319-B27] Pérez P , de los Campos G. Genome-wide regression and prediction with the BGLR statistical package. Genetics. 2014;198(2):483–495. doi:10.1534/genetics.114.164442. 25009151 PMC4196607

[jkac319-B28] Pritchard JK , Di Rienzo A. Adaptation—not by sweeps alone. Nat Rev Genet. 2010;11(10):665–667. doi:10.1038/nrg2880. 20838407 PMC4652788

[jkac319-B29] Pritchard JK , Pickrell JK, Coop G. The genetics of human adaptation: hard sweeps, soft sweeps, and polygenic adaptation. Curr Biol. 2010;20(4):R208–R215. doi:10.1016/j.cub.2009.11.055. 20178769 PMC2994553

[jkac319-B30] Racimo F , Berg JJ, Pickrell JK. Detecting polygenic adaptation in admixture graphs. Genetics. 2018;208(4):1565–1584. doi:10.1534/genetics.117.300489. 29348143 PMC5887149

[jkac319-B31] R: The R Project for Statistical Computing,

[jkac319-B32] Sargolzaei M , Schenkel FS. QMSim: a large-scale genome simulator for livestock. Bioinformatics. 2009;25(5):680–681. doi:10.1093/bioinformatics/btp045. 19176551

[jkac319-B33] Sohail M , Maier RM, Ganna A, Bloemendal A, Martin AR, Turchin MC, Chiang CWK, Hirschhorn J, Daly MJ, Patterson N, Neale B, Mathieson I, Reich D, Sunyaev S. Polygenic adaptation on height is overestimated due to uncorrected stratification in genome-wide association studies. eLife. 2019;8:e39702. doi:10.7554/eLife.39702. 30895926 PMC6428571

[jkac319-B34] Turchin MC , Chiang CW, Palmer CD, Sankararaman S, Reich D, GIANT Consortium, Hirschhorn J. Evidence of widespread selection on standing variation in Europe at height-associated SNPs. Nat Genet. 2012;44(9):1015–1019. doi:10.1038/ng.2368. 22902787 PMC3480734

[jkac319-B35] Visscher PM , Hemani G, Vinkhuyzen AAE, Chen G-B, Lee SH, Wray NR, Goddard ME, Yang J. Statistical power to detect genetic (co)variance of complex traits using SNP data in unrelated samples. PLoS Genet. 2014;10(4):e1004269. doi:10.1371/journal.pgen.1004269. PMC3983037 24721987

[jkac319-B36] Visscher PM , Wray NR, Zhang Q, Sklar P, McCarthy MI, Brown MA, Yang J. 10 Years of GWAS discovery: biology, function, and translation. Am J Hum Genet. 2017;101(1):5–22. doi:10.1016/j.ajhg.2017.06.005. 28686856 PMC5501872

[jkac319-B37] Voss-Fels KP , Stahl A, Wittkop B, Lichthardt C, Nagler S, Rose T, Chen TW, Zetzsche H, Seddig S, Baig MM, Ballvora A, Frisch M, Ross E, Hayes BJ, Hayden MJ, Ordon F, Leon J, Kage H, Friedt W, Stützel H, Snowdon RJ. Breeding improves wheat productivity under contrasting agrochemical input levels. Nat Plants. 2019;5(7):706–714. doi:10.1038/s41477-019-0445-5. 31209285

[jkac319-B38] Wood AR , Esko T, Yang J, Vedantam S, Pers TH, et al Defining the role of common variation in the genomic and biological architecture of adult human height. Nat Genet. 2014;46(11):1173–1186. doi:10.1038/ng.3097. 25282103 PMC4250049

[jkac319-B39] Wright S . Evolution in Mendelian populations. Genetics. 1931;16(2):97–159. doi:10.1093/genetics/16.2.97. 17246615 PMC1201091

[jkac319-B40] Zeng J , de Vlaming R, Wu Y, Robinson MR, Lloyd-Jones LR, Yengo L, Yap X, Xue A, Sidorenko J, McRae AF, Powell JE, Montgomery GW, Metspalu A, Esko T, Gibson G, Wray NR, Visscher PM, Yang J. Signatures of negative selection in the genetic architecture of human complex traits. Nat Genet. 2018;50(5):746–753. doi:10.1038/s41588-018-0101-4. 29662166

