# Supplementary material for: Ghat: an R package for identifying adaptive polygenic traits
Source: G3 (Bethesda). 2022 Dec 1;13(2):jkac319. doi: 10.1093/g3journal/jkac319 (PMC9911052; doi:10.1093/g3journal/jkac319)
Supplement: jkac319_Supplementary_Data [file jkac319_supplementary_data.docx]

**Supplementary table 1**. Objects, data, functions, and options found in Ghat package.

| **Function** | | **Description** |
| --- | --- | --- |
| Ghat (Function) | effects | Input vector of allele substitutional effects. |
|  | change | Input vector of changes in allele frequency between two different generations (could be positive, negative or zero). |
|  | method | One of "vanilla" (assumes complete linkage equilibrium between markers), "trim" (excludes markers to approximate linkage equilibrium some of the extreme values), or "scale" (scales results to reflect underlying levels of linkage LD). The default value is "scale". |
|  | perms | Input number to set the number of permutations to test for significance. The default value is 1000. |
|  | plot | Input option to select which plot you need to be returned: "Ghat", "Cor", or "Both". The default value is "Ghat". |
|  | blockSize | Input number to set the size of the window on each chromosome for trimming, only required if method = "trim". No default. |
|  | num_eff | Input number to set the effective number of independent markers, only required if method = " scale". Can be calculated using “ld_decay” function above. No default. |
| ld_decay  (Function) | gen | Input matrix of genotype data. Individuals in rows, genotypes (0, 1, 2) in columns. |
|  | map | Input data frame including the name for each marker with a corresponding chromosome number and physical position. |
|  | max_win_snp | Input number to set the maximum number of markers allowed per window within a chromosome before estimating the LD. The default value is 2000. |
|  | max.chr | Input number to select to which chromosome you want to run the analysis, chromosomes above this number will be excluded from the analysis. No default. |
|  | cores | Input number to set the optimal number of cores for using parallelized calculation, since calculation of LD require too much memory. The default value is 1. |
|  | max_r2 | Input number to set the threshold of correlation between the neighbourhood variant (r^2^) to calculate the effective number of independent variants. No default. |
| Maize_wqs (Data object) | Maize_wqs[[1]] | Data frame, including all SNP names (SNP Names), effects on Acid detergent fiber (adf) (effects. adf), and changes in allele frequencies (change) between cycle 1 and cycle 3 of selection. |
|  | Maize_wqs[[2]] | Data frame, including plant ID’s (taxa), estimated breeding values for Acid detergent fiber (adf), and generation when the phenotyped strains were grown (year). |
|  | Maize_wqs[[3]] | Data frame, map file, each line of the Map file describes a single marker and contain three columns. 1: Name (SNP id); 2: Chromosome (Chromosome number); 3: SNP position (in base-pairs). |
|  | Maize_wqs[[4]] | Data frame, Genotype (Illumina MaizeSNP50 BeadChip); an Infinium HD assay (Illumina, Inc. San Diego, CA). 10,017 SNP markers (0, 1 and 2) after filtration, distributed across the maize genome [43]. |


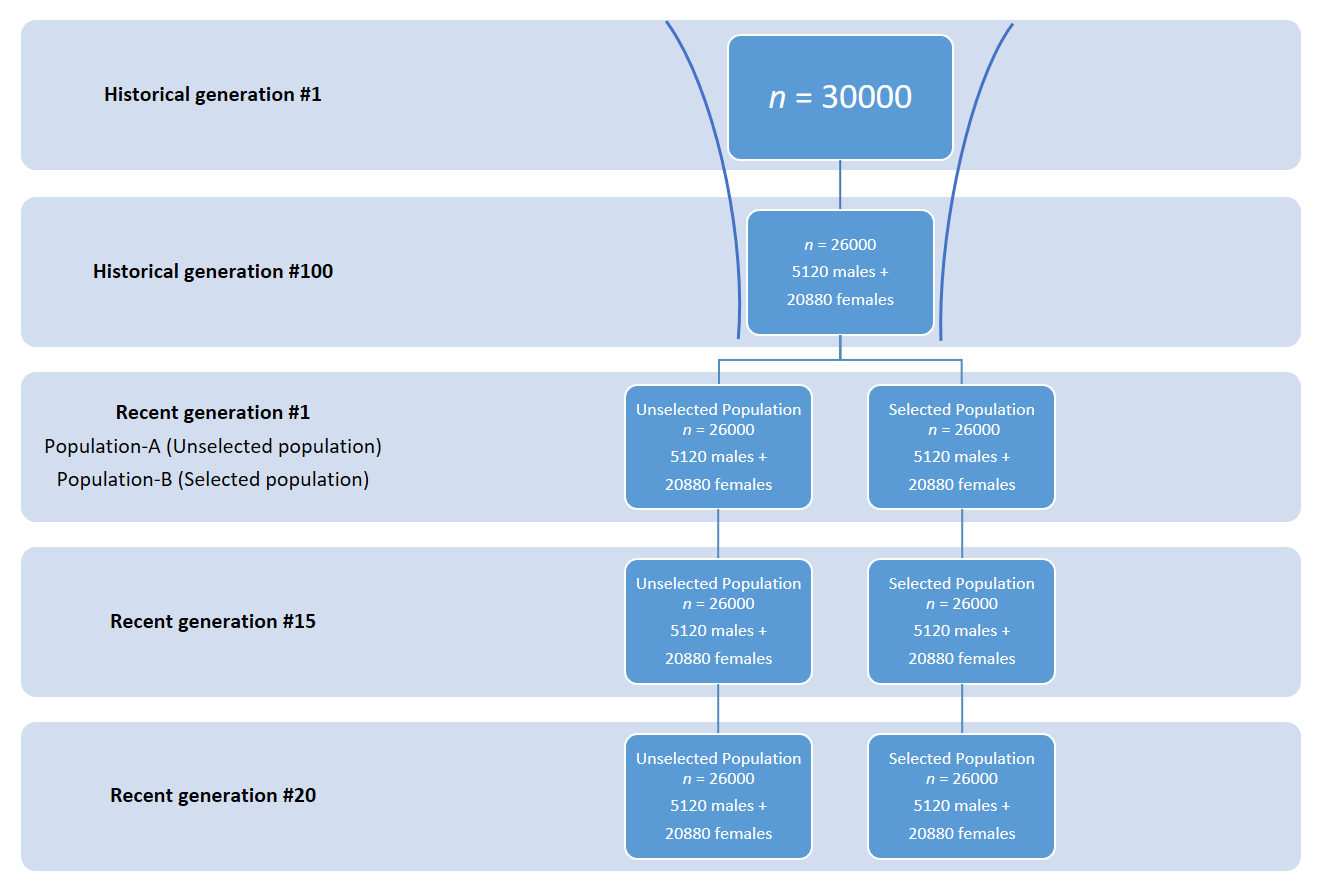


**Supplementary Figure 1**. QMSim simulation scheme. This figure depicts how data were simulated for analysis with Ghat. Each branch in the figure depicts a population. 100 historical generations without selection were simulated, followed by 20 recent generations that included selection. No selection operated on Population-A, the unselected population, and in Population-B, the selected population, animals were selected based on their simulated phenotypic value. Population size remained constant during all 20 recent generations. Data from recent generation number 15 and 20 were used for analysis.

| **A-**  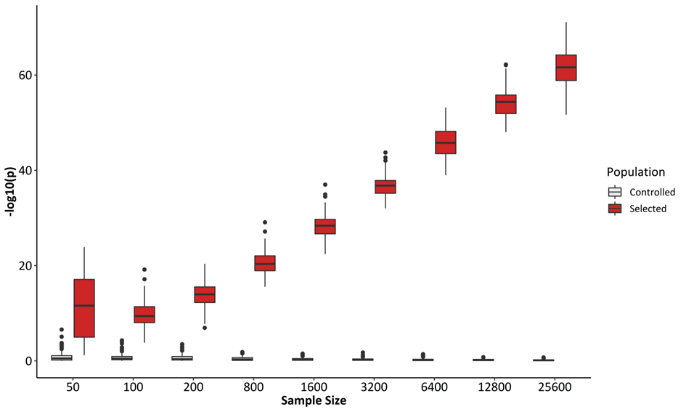 | **B-**  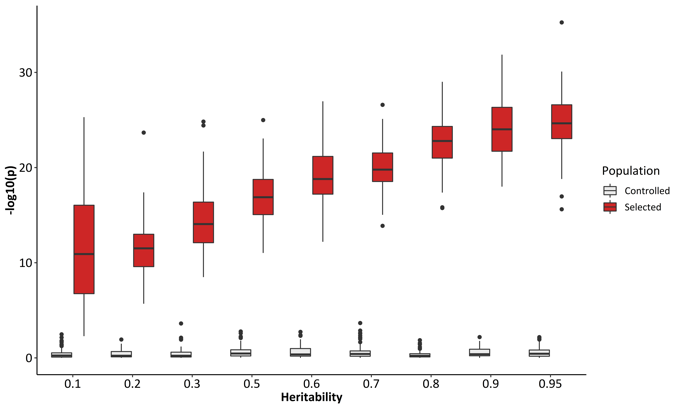 |
| --- | --- |
| **C-**  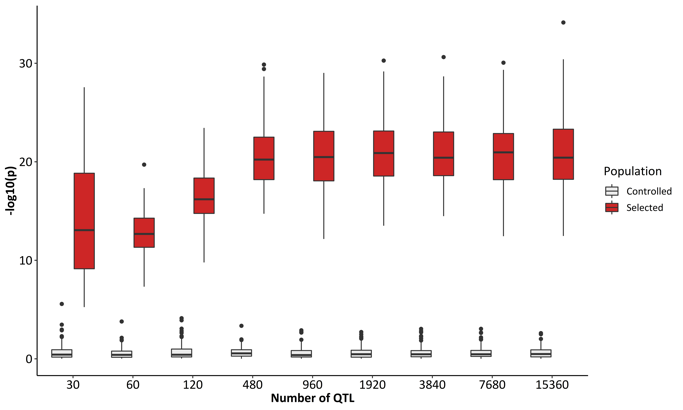 | **D-**  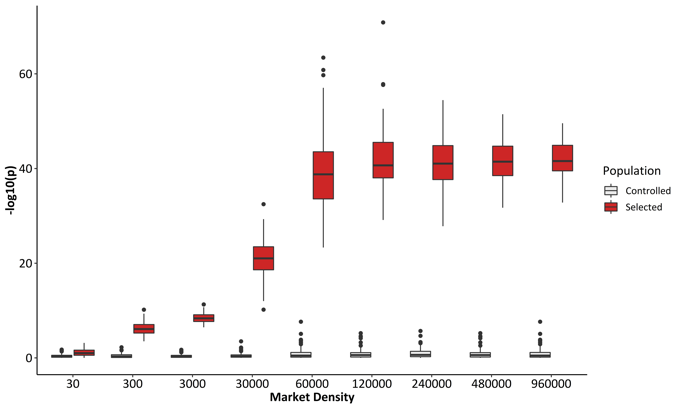 |

**Supplementary Figure 2 A- The effect of *n* on Ghat.** Results depict 1000 replications testing the effect of *n* on Ghat-P.values, white corresponds to unselected (control) populations and red corresponds to selected populations. **B-** **The effect of *h^2^* on Ghat.** Results depict the effect of *h^2^* on the power of Ghat. White indicates the range of 1000 unselected (control) population replicates and red indicates the range of 1000 selected population replicates. **C-** **The effect of *nQTL* in the Ghat-test for selection.** Results are for 1000 replications testing the effect of *nQTL* on Ghat-P.values, white corresponds to unselected (control) populations and red corresponds to selected populations. **D- The effect of *MD* in Ghat-tests for selection.** Results depict 1000 replicated simulations testing the effect of *MD* on the power of Ghat. White corresponds to no-selection (control) simulations and red corresponds to selection simulations.
